# Correction: ATP hydrolysis by the viral RNA sensor RIG-I prevents unintentional recognition of self-RNA

**DOI:** 10.7554/eLife.14954

**Published:** 2016-03-02

**Authors:** Charlotte Lässig, Sarah Matheisl, Konstantin MJ Sparrer, Carina C de Oliveira Mann, Manuela Moldt, Jenish R Patel, Marion Goldeck, Gunther Hartmann, Adolfo García-Sastre, Veit Hornung, Karl‐Klaus Conzelmann, Roland Beckmann, Karl-Peter Hopfner

Lässig C, Matheisl S, Sparrer KMJ, de Oliveira Mann CC, Moldt M, Patel JR, Goldeck M, Hartmann G, García-Sastre A, Hornung V, Conzelmann K-K, Beckmann R, Hopfner K-P. 2015. ATP hydrolysis by the viral RNA sensor RIG-I prevents unintentional recognition of self-RNA. *eLife* **4**:e10859. doi: 10.7554/eLife.10859.

Published November 26, 2016

We identified an error in Figure 3B where the labeling of RIG-I and E373Q was incorrectly switched. During the revision of our figures we introduced this mistake and now switched the experimental figures according to the labeling.

The corrected Figure 3 is also shown here:

**Figure:**
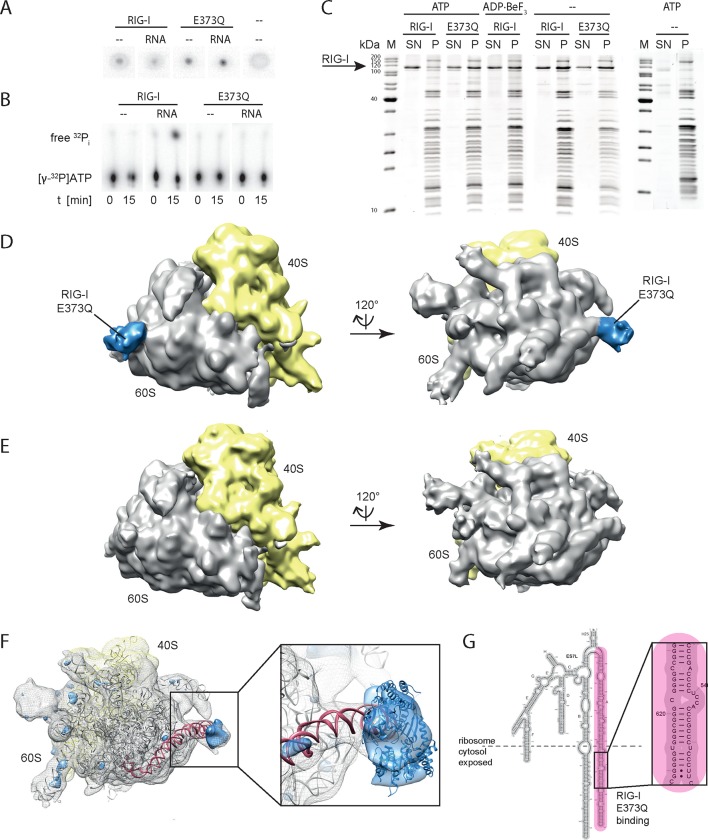

The originally published Figure 3 is also shown for reference:

**Figure:**
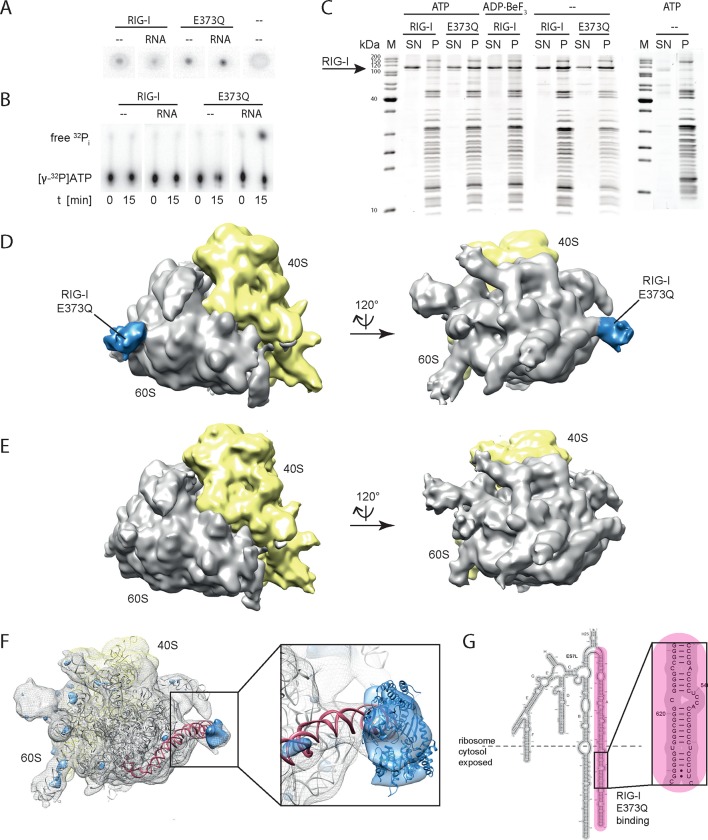

The article has been corrected accordingly.

